# Computed Tomography of Neoplastic Infiltrating Renal Masses in Patients Without a Previous History of Cancer

**DOI:** 10.3390/cancers17172936

**Published:** 2025-09-08

**Authors:** Carlos Nicolau, Andreu Ivars, Carmen Sebastia, Clara Bassaganyas, María Fresno, Leonardo Rodríguez, Josep Puig, Marc Comas-Cufí, Blanca Paño

**Affiliations:** 1 Radiology Department, Clinic Hospital, IDIBAPS, University of Barcelona (UB), 08036 Barcelona, Spain; ivars@clinic.cat (A.I.); msebasti@clinic.cat (C.S.); bassaganyas@clinic.cat (C.B.); mfresno@clinic.cat (M.F.); lerodrig@clinic.cat (L.R.); jpuig2@clinic.cat (J.P.); bpano@clinic.cat (B.P.); 2 Department of Computer Science, Applied Mathematics and Statistics, University of Girona, 17071 Girona, Spain; marc.comas@udg.edu

**Keywords:** infiltrative renal masses, computed tomography (CT), renal cell carcinoma (RCC), urothelial carcinoma (UC), renal lymphoma, radiologic features

## Abstract

Background/Objectives: Infiltrative renal masses, characterized by ill-defined margins and parenchymal invasion without forming a discrete mass, present a diagnostic challenge, particularly in patients without a prior history of malignancy. Differentiating among the most common malignant etiologies—renal cell carcinoma (RCC), urothelial carcinoma (UC), and lymphoma—is essential for guiding appropriate treatment. This study aimed to evaluate whether specific computed tomography (CT) features can assist in the differential diagnosis of these lesions. Methods: A retrospective review was conducted on 68 patients with infiltrative renal masses presented at a tertiary hospital’s oncologic urology committee between 2018 and 2022. Patients with prior malignancy or signs of infection were excluded. All cases underwent contrast-enhanced CT within three months of diagnosis and had histopathological confirmation. Imaging features such as necrosis, collecting system involvement, lymphadenopathy, and others were assessed and statistically analyzed. Results: RCC was the most frequent diagnosis (68%), followed by UC (18%) and lymphoma (7.4%). Significant differences were observed in imaging features: necrosis was more common in RCC (87%) than in UC (25%) and lymphoma (20%), *p* < 0.001; collecting system involvement was universal in UC (100%) and less common in RCC (65%) and lymphoma (40%), *p* = 0.009; and lymphadenopathy was more frequent in lymphoma (80%) than in UC (67%) and RCC (35%), *p* = 0.038. Tumor size also varied significantly, with lymphomas presenting the largest median size (11 cm), followed by RCCs (8.2 cm) and UCs (5 cm), *p* < 0.001. Conclusions: CT imaging features, particularly necrosis, collecting system involvement, and lymphadenopathy, can aid in distinguishing among RCC, UC, and lymphoma in patients with infiltrative renal masses and no prior cancer history. These findings may support more accurate diagnoses and inform tailored therapeutic strategies.

## Simple Summary

Infiltrative renal masses pose a diagnostic challenge due to their nonspecific imaging features and broad spectrum of malignant etiologies. This retrospective study aimed to assess whether specific computed tomography (CT) findings could aid in differentiating among the most common diagnoses in patients without a prior history of malignancy or signs of infection. Radiologic features such as intratumoral necrosis, collecting system involvement, and lymphadenopathy were identified as significant discriminators, helping in the differential diagnosis between renal cell carcinoma, urothelial carcinoma, and lymphoma. These findings may support a more accurate and timely diagnostic approach, ultimately improving therapeutic decision-making in complex clinical scenarios.

## 1. Introduction

Infiltrative renal masses characterized by interstitial proliferation of cells around nephrons, collecting ducts, and blood vessels typically present with ill-defined margins and demonstrate a propensity to invade the renal parenchyma without forming a discrete, well-circumscribed mass [1]. Most infiltrative renal masses represent primary or secondary malignancies with poor prognosis [2,3], but certain benign conditions—such as acute pyelonephritis, xanthogranulomatous pyelonephritis, and renal sarcoidosis—can also present with an infiltrative pattern [4,5,6]. Inflammatory or infectious etiologies are typically associated with systemic symptoms such as fever and supportive laboratory findings.

In patients with a known history of malignancy, metastases are the most common cause. In those without a prior cancer diagnosis, the most frequent malignant etiologies include aggressive subtypes of renal cell carcinoma (RCC)—such as collecting duct carcinoma, medullary carcinoma, and sarcomatoid RCC—urothelial carcinoma (UC), and renal lymphoma [4,7]. A prior series reported that approximately 45% of infiltrative renal masses were RCCs, 30% represented urothelial carcinoma, followed by 10% lymphoma, 10% metastatic disease to the kidney, and 5% other etiologies, including inflammatory or infectious processes [2]. Distinguishing between RCC, UC, and lymphoma can be challenging but is critical for appropriate management [2,8], as treatment strategies differ significantly, ranging from nephrectomy versus systemic therapy for infiltrative RCC, nephroureterectomy versus systemic therapy for UC, and systemic treatment alone in the case of lymphoma.

Cross-sectional imaging, particularly computed tomography (CT) and magnetic resonance imaging (MRI), plays a pivotal role in the characterization of infiltrating renal masses, as it can show features such as heterogeneous, ill-defined enhancement, loss of corticomedullary differentiation, and preservation of the renal contour despite extensive parenchymal involvement [9,10,11,12]. In advanced cases, perinephric extension or vascular invasion may also be observed. Previous literature has addressed the radiological features of aggressive RCC and UC, with particular emphasis on differentiating these entities using imaging techniques including CT [7,13,14,15] and MRI [16,17]. In the study of Chen X, hydronephrosis, heterogeneous enhancement, and preserving reniform contour were independent factors in distinguishing between UC presenting as solid renal masses and RCC with collecting system invasion. Moreover, emerging evidence underscores the diagnostic potential of radiomics in differentiating these tumors, with promising results reported by authors such as Zhai X and Marcon J [18,19].

The aim of this study was to evaluate whether CT imaging features can assist in the differential diagnosis of infiltrative renal masses in patients without a prior history of malignancy.

## 2. Materials and Methods

Patients.

A retrospective study was conducted on indeterminate infiltrative renal masses presented at our hospital’s oncologic urology committee over the past seven years.

A retrospective search for infiltrative renal tumors was conducted among cases presented at the multidisciplinary oncologic urology tumor board of Hospital Clinic between January 2018 and December 2022. From this cohort, tumors in patients with a known history of cancer or with clinical or laboratory findings suggestive of infection were excluded. The included infiltrative tumors were defined as renal masses with ill-defined borders relative to the adjacent parenchyma, and all infiltrative patterns were confirmed by two radiologists (AI, CB) who reviewed the imaging studies.

Inclusion criteria were as follows:

- Presence of at least one renal mass with an infiltrative appearance in patients without a prior history of malignancy and without clinical or laboratory evidence of infection.
- A contrast-enhanced abdominal CT scan performed in the 3 months prior to establishing a final diagnosis.
- Histopathological confirmation of the final diagnosis.

CT examinations.

All studies were performed with multidetector CT equipment (Siemens Healthcare, Erlangen, Germany) with slice thickness of 2–5 mm for review. Each patient received 80–100 mL of intravenous iodinated contrast medium (iohexol 240). We used one of the following protocols depending on the clinical indication: The first was applied for general abdominal indications such as abdominal pain or suspected abdominal masses and included a portal venous phase acquired at 70 s and an excretory phase starting at 180 s following intravenous contrast administration. The second protocol was specifically designed for renal lesion characterization and included four phases: an unenhanced phase, a corticomedullary phase at 35 s, a nephrographic phase at 90 s, and an excretory phase beginning at 180 s after contrast injection. Coronal reformatted images were also obtained in all cases. Various imaging features of the renal lesions were selected and evaluated by the same group of radiologists (AI, CB), including lesion number, size, and the presence or absence of the following signs: calcifications, necrosis, renal sinus involvement, collecting system involvement, perirenal fat invasion, renal vein thrombosis, lymphadenopathy, and metastatic disease.

These signs were defined as follows:

- Calcifications were defined as hyperdense foci within the lesion, with attenuation values significantly higher than the surrounding soft tissue, typically exceeding 100 Hounsfield Units (HUs), and visible in non-contrast CT images.
- Intratumoral necrosis was defined as the presence of hypodense areas within the tumor lacking a defined wall and showing no enhancement in any of the imaging phases (corticomedullary, portal or nephrographic, or excretory).
- Perirenal fat invasion was defined as irregular tumor margins breaching the renal capsule, accompanied by stranding or nodularity within the adjacent perirenal fat.
- Renal sinus involvement was defined as irregular tumor margins extending into the sinus fat, with associated stranding or nodularity within the renal sinus.
- Tumoral venous thrombosis was defined as a filling defect within the vein that exhibits enhancement similar to that of the primary tumor.
- Lymphadenopathy was defined as the presence of enlarged retroperitoneal lymph nodes measuring greater than 10 mm in short-axis diameter.

Additional imaging features such as multiplicity (presence of more than one infiltrative lesion) and bilaterality (involvement of both kidneys) were also recorded and analyzed. All imaging features were reviewed by a senior radiologist (CN), who validated the findings. In cases of discrepancy, final decisions were reached by consensus. For cases with multiple lesions, the largest lesion was selected for statistical analysis.

### Statistical Analysis

Quantitative variables are expressed as the median (IQR), and categorical variables are expressed as numbers (percentage). Patients were divided into groups based on the histology, but only those lesions representing more than 3% of the total number of cases were included in the statistical analysis. Demographic data and lesion characteristics of the groups were compared. For numerical variables, we applied the Welch’s ANOVA test in the absence of normality and the Kruskal–Wallis test when normality was assumed. For categorical variables, Pearson’s chi-square test was used when expected cell counts were sufficient; otherwise, Fisher’s exact test was applied. A *p*-value <0.05 was considered statistically significant. All statistical analyses were performed using R (version 4.5.0).

## 3. Results

A total of 68 patients with infiltrating renal masses were included in this study. Table 1 shows the demographic and radiological characteristics of the patients.

Histological diagnosis was obtained through nephrectomy in 51 patients and via percutaneous biopsy in 17 patients.

Fifty-eight patients (85%) had a single lesion in one kidney, and ten (14.5%) had multiple lesions: nine patients (13%) in one kidney and one patient (1.5%) in both kidneys.

**Figure 1:**
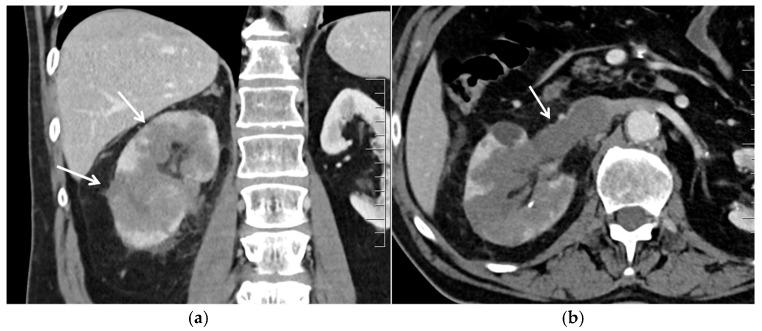
Infiltrating right clear cell RCC with renal vein thrombosis. (**a**) Contrast-enhanced coronal CT image in the venous phase demonstrates a hypoenhancing infiltrative lesion (arrows) with an ill-defined interface with the adjacent normal renal parenchyma. The reniform contour of the kidney is preserved. (**b**) Contrast-enhanced axial CT image in the venous phase shows tumor extension into the right renal vein (arrow), reaching the inferior vena cava.

The most frequent diagnosis among infiltrative renal masses was renal cell carcinoma (46 cases, 68%) (Figure 1), followed by urothelial carcinoma (12 cases, 18%) and lymphoma (5 cases, 7.4%). According to the WHO/ISUP grading system for clear cell RCC and papillary RCC, among the 46 RCCs, 33 were classified as clear cell carcinomas (2 grade 2, 8 grade 3, and 23 grade 4). Eight were classified as papillary type 2 RCCs (seven grade 4 and one grade 3), two were classified as chromophobe RCCs, and three were classified as undifferentiated RCC.

Less common etiologies included neuroendocrine tumors (2.9%), liposarcoma (1.5%), schwannoma (1.5%), and yolk sac tumor (1.5%). Together, these rare diagnoses accounted for 7.4% of cases.

**Figure 2:**
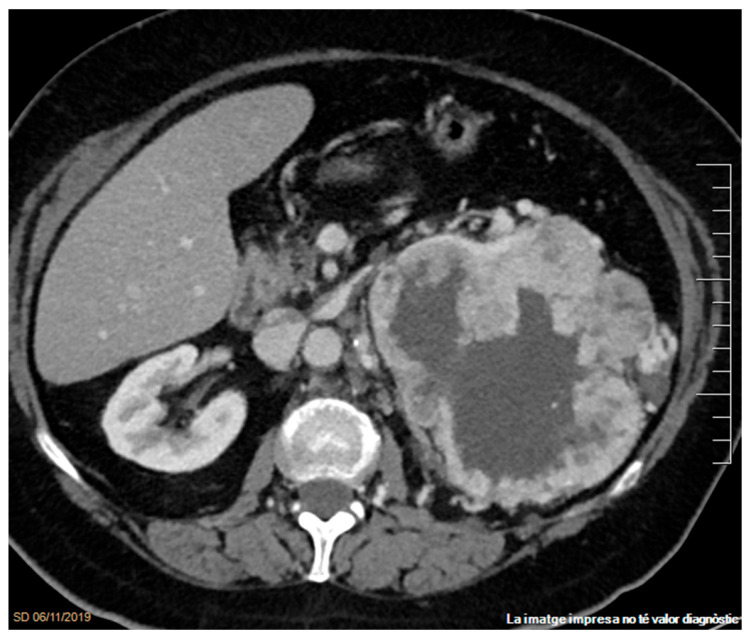
Infiltrating left clear cell RCC with necrosis. Contrast-enhanced axial CT image in the venous phase demonstrates a hypervascular mass with heterogeneous enhancement, attributable to a large non-enhancing necrotic area. Peritumoral vessels are visible within the perirenal space.

**Figure 3:**
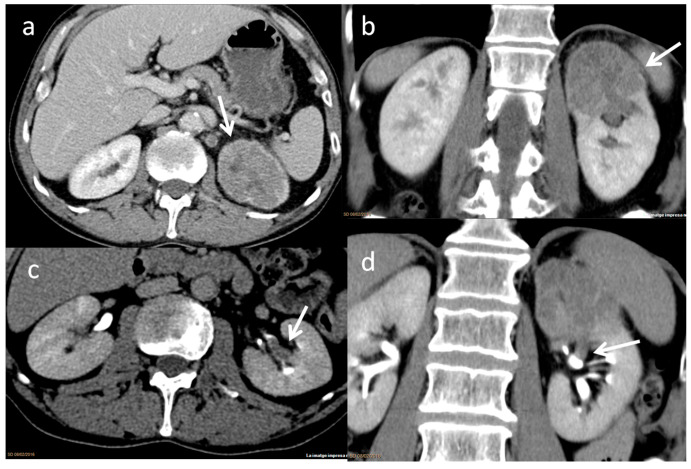
Infiltrating UC with parenchymal involvement. Contrast-enhanced nephrographic phase axial (**a**) and coronal (**b**) CT images demonstrate a diffusely infiltrative tumor involving the renal cortex and sinus of the upper pole of the left kidney (arrow), with preservation of the overall renal contour. Contrast-enhanced excretory phase axial (**c**) and coronal (**d**) images show tumor extension into the excretory tract, resulting in a focal filling defect (arrow).

The most common radiological findings of the infiltrating renal masses were renal sinus involvement (in 78% of the tumors), intratumoral necrosis (68%) (Figure 2), and collecting system involvement (69%) (Figure 3).

**Figure 4:**
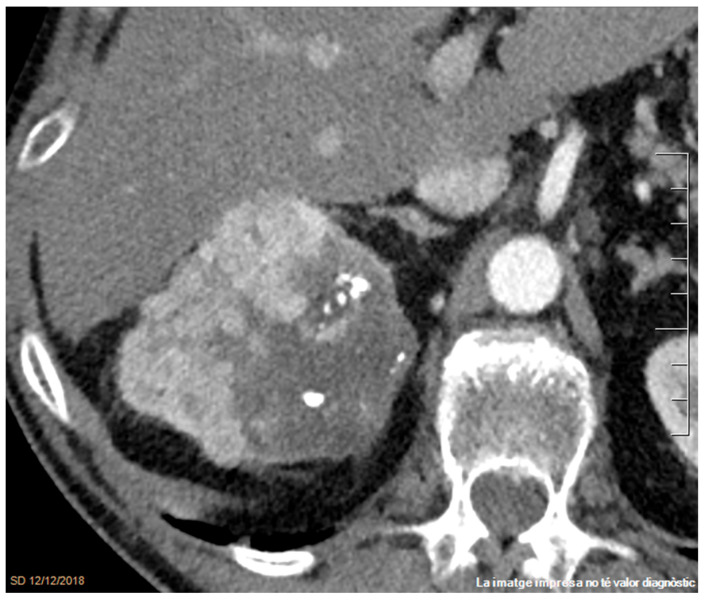
Infiltrating right clear cell RCC with calcifications. A contrast-enhanced axial CT image in the venous phase demonstrates a hypervascular mass with calcifications and heterogeneous enhancement due to the presence of a large non-enhancing necrotic area. Peritumoral vessels are visible within the perirenal space.

On the contrary, the presence of intratumoral calcifications was found in only 19% (Figure 4) and venous thrombosis in 31% of the masses.

Table 2 presents a comparison of the radiological features among the three most common diagnoses: RCC, UC, and lymphoma.

**Figure 5:**
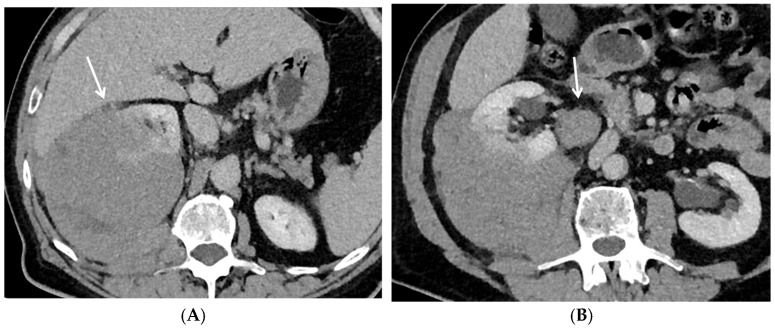
Lymphoma involving the right renal parenchyma. Contrast-enhanced nephrographic phase axial CT images (**A**,**B**) show a large infiltrative, homogeneously enhancing mass in the right kidney (arrow, **A**), with extension into the perirenal space (white arrow, **B**). Associated lymphadenopathy is noted in the renal hilum (arrow, **B**).

Regarding the imaging features analyzed, tumor size significantly differed among the groups, with lymphoma showing the largest median size; UC was strongly associated with excretory tract involvement; necrosis was markedly more frequent in RCC; and lymphomas showed the highest rate of lymphadenopathy (Figure 5). Although the difference in calcification prevalence among tumor types did not reach statistical significance (*p* = 0.08), the trend suggests a potential diagnostic role, as they were found in 26% of renal RCC, whereas no calcifications were observed in lymphomas or UCs.

## 4. Discussion

Infiltrative renal masses represent a diagnostic challenge due to their nonspecific imaging characteristics and broad differential diagnosis, particularly in patients without a prior history of malignancy. This study aimed to evaluate whether CT imaging features could aid in distinguishing among the most common etiologies—RCC, UC, and lymphoma—in this patient population.

Our findings confirm that RCC is the most prevalent cause of infiltrative renal masses in patients without a known malignancy, accounting for more than two-thirds of cases (68%). UC and lymphoma are the next most common, comprising 18% and 7.4% of cases, respectively, in our series. Collectively, these three entities comprise 92.6% of infiltrative renal lesions. These results are consistent with previous literature, which identifies aggressive RCC subtypes and urothelial tumors as leading malignant causes of infiltrative renal lesions in the absence of systemic symptoms or infection. In a similar study led by Dr. Wang, where the inclusion criterion was the presence of a radiologically documented infiltrative renal lesion, the most frequent diagnoses, in descending order of prevalence, were RCC, UC, lymphoma, and metastases [2].

Several imaging features demonstrated statistically significant differences among tumor types, suggesting their potential utility in narrowing the differential diagnosis.

Infiltrating RCCs with invasive growth patterns are usually aggressive subtypes—such as collecting duct carcinoma, medullary carcinoma, and sarcomatoid RCC—with imaging showing heterogeneous enhancement and parenchymal distortion without a discrete mass [10]. In these aggressive subtypes, the presence of intertumoral necrosis and extension of the tumor into the renal vein are common.

Regarding the presence of tumoral necrosis, it is a frequent histopathological finding in RCC and has been extensively studied for its prognostic significance [20]. According to the WHO classification and recent literature, the presence of necrosis is associated with more aggressive RCC subtypes, particularly clear cell RCC [21]. Intratumoral macroscopic necrosis has been previously identified as an independent marker of aggressiveness in renal cell carcinomas (RCCs). RCCs with macroscopic necrosis were more likely to present with larger tumor size, metastatic disease, higher local stage, and higher tumor grade. Moreover, patients with macroscopic tumor necrosis demonstrated significantly worse disease-specific survival. Necrosis can also be observed in other malignant renal tumors, including UC and lymphoma, but it is more common in RCCs, as happened in our study, being significantly more common in RCC (87%) compared to UC (25%) and lymphoma (20%) (*p* < 0.001), reflecting the aggressive and heterogeneous nature of infiltrating RCC.

Regarding the presence of renal vein thrombosis, cancer is associated with a high risk of venous thrombosis, and renal veins can show thrombi that sometimes extend into the vena cava. Renal vein thrombosis is much more common in RCC but can also be seen in other tumors such as UC, leiomyosarcomas, or lymphomas, as was previously described in the literature [22,23,24].

Another investigated feature, the presence of calcifications, was not statistically significant (*p* = 0.08), but calcifications were exclusively observed in RCC (26%), suggesting a potential, albeit limited, role in differentiating RCC from other infiltrative neoplasms, particularly in ambiguous cases. Due to the limited sample size and lack of statistical significance, calcifications should not be used in isolation for diagnostic decision-making but rather integrated with other radiologic and clinical findings (such as necrosis, tumor size, and excretory tract involvement). Consistent with our findings, the presence of calcifications in solid renal masses has been associated with renal cell carcinoma (RCC) in the literature [25].

Other findings, such as renal sinus involvement and perirenal fat invasion, were common across all tumor types, limiting their discriminative value. In addition, metastatic disease was observed in a substantial proportion of RCC cases but did not reach statistical significance when compared across groups, as all infiltrating cancers are aggressive.

Regarding UC, they typically arise from the renal pelvis, infiltrating the kidney while preserving its architecture, often accompanied by hydronephrosis or delayed contrast washout [14,26]. Hence, the excretory tract involvement was a hallmark of UC, present in 100% of cases, compared to 65% in RCC and 40% in lymphoma (*p* = 0.009). Although involvement of the excretory tract does not allow for a definitive distinction between UC and RCC, the presence of additional findings such as necrosis, calcifications, or renal vein thrombosis points towards a diagnosis of RCC [3,4]. Furthermore, the absence of excretory tract involvement may help exclude the diagnosis of UC.

Finally, findings more common in lymphomas were a big size of the tumor and the presence of enlarged lymph nodes. In our study, tumor size varied significantly, with lymphomas presenting with the largest median size (11 cm), followed by RCC (8.2 cm) and UC (5 cm) (*p* < 0.001). This aligns with the known biology of renal lymphoma, which often presents as large, homogeneous masses with minimal necrosis. Renal lymphoma may be secondary to hematogenous dissemination or to contiguity of retroperitoneal lymphadenopathies [27]. Primary isolated renal lymphoma is very rare (˂1% of all extranodal lymphomas). Renal lymphomatous involvement may present as multiple focal masses, large infiltrative lesions, or diffuse bilaterally enlarged kidneys [9,23,28]. Other findings that support the diagnosis of lymphoma are concomitant bulky lymphadenopathy and bilateral involvement [1]. In our series, the presence of lymphadenopathy was most frequently observed in lymphoma (80%) and UC (67%), compared to 35% in RCC (*p* = 0.038), which may reflect the systemic nature of lymphoma and the lymphotropic behavior of urothelial carcinoma.

The combination of CT findings and the integration of clinical and imaging data can assist in the management of infiltrative renal masses, as previously described [3,4]. Specifically, the presence of a solitary infiltrative renal mass associated with hematuria and involvement of the excretory tract suggests UC, although RCC cannot be ruled out. A solitary infiltrative mass with necrosis, calcifications, or renal vein thrombosis is more suggestive of RCC. In contrast, lymphoma typically presents as multiple infiltrative renal masses with associated lymphadenopathy.

Furthermore, there is broad consensus regarding the inclusion of renal biopsy in the diagnostic algorithms for infiltrative renal masses when the results may influence clinical decision-making [29]. There is also active research into the use of advanced imaging techniques in this context, such as radiomics and PET-CT. For example, when the differential diagnosis lies between renal lymphoma and RCC, PET-CT can be a valuable tool, as renal lymphoma tends to be intensely FDG-avid, whereas RCC may exhibit low or variable FDG uptake [30].

This study has several limitations. First, its retrospective design and relatively small sample size—particularly concerning less prevalent tumor subtypes—may limit the generalizability of the results. Second, the patient cohort was exclusively derived from the oncologic urologic tumor board of our institution, which may introduce selection bias. Another limitation is the lack of integration of clinical and imaging data in the statistical analysis. Furthermore, individuals with clinical or laboratory evidence of infection were excluded, potentially narrowing the spectrum of benign conditions that could mimic malignancy and thus limiting the comprehensiveness of the differential diagnostic analysis.

## 5. Conclusions

In conclusion, our preliminary results suggest that certain CT features—particularly necrosis, excretory tract involvement, and lymphadenopathy—can provide valuable clues in the differential diagnosis of infiltrative renal masses. These imaging characteristics, when interpreted in conjunction with clinical context, may help guide further diagnostic workup and inform treatment planning. Collaborative decision-making with urologists and oncologists is essential, as treatment ranges from nephrectomy for localized infiltrative RCC to systemic therapy for disseminated disease. Due to the small sample size of this preliminary study, further research, including multicenter studies with larger cohorts, is needed to validate our findings and explore utility in clinical decision-making.

## Figures and Tables

**Table 1:** Demographic, clinical, and radiological characteristics of patients with infiltrative renal masses (N = 68).

Variable	Value
Age (years)	67 (59, 75)
Gender (Female)	20 (29%)
Lesion Number	
• Single lesion	58 (85%)
• Bilateral multiplicity	1 (1.5%)
• Unilateral multiplicity	9 (13%)
Laterality	
• Right	38 (56%)
• Left	29 (43%)
• Graft	1 (1.5%)
Final Diagnosis	
• Renal carcinoma	46 (68%)
• Liposarcoma	1 (1.5%)
• Lymphoma	5 (7.4%)
• Neuroendocrine tumor	2 (2.9%)
• Schwannoma	1 (1.5%)
• Yolk sac tumor	1 (1.5%)
• Urothelial carcinoma	12 (18%)
Tumor Size (cm)	7.9 (6.4, 10)
Imaging Features	
• Calcifications	13 (19%)
• Renal sinus involvement	53 (78%)
• Collecting system involvement	47 (69%)
• Necrosis	46 (68%)
• Perirenal fat invasion	40 (59%)
• Renal vein thrombosis	21 (31%)
• Lymphadenopathy	28 (41%)
• Metastatic disease	27 (40%)

**Table 2:** Radiological features of the most common infiltrative lesions.

Characteristics	RCC N = 46	Lymphoma N = 5	UC N = 12	*p*-Value
Age	65 (58, 75)	72 (65, 75)	69 (64, 77)	0.4
Female gender	15 (33%)	1 (20%)	1 (8.3%)	0.3
Number of lesions				0.8
One	39 (85%)	4 (80%)	10 (83%)	
Multiple bilateral	1 (2.2%)	0 (0%)	0 (0%)	
Multiple unilateral	6 (13%)	1 (20%)	2 (17%)	
Laterality				0.12
Right	28 (61%)	2 (40%)	5 (42%)	
Left	18 (39%)	2 (40%)	7 (58%)	
Renal transplant	0 (0%)	1 (20%)	0 (0%)	
Size (cm)	8.2 (7, 10)	11 (6.7, 20)	5 (3.7, 6.5)	<0.001
Calcifications	12 (26%)	0 (0%)	0 (0%)	0.083
Renal sinus involvement	35 (76%)	4 (80%)	11 (92%)	0.6
Excretory tract involvement	30 (65%)	2 (40%)	12 (100%)	0.009
Necrosis	40 (87%)	1 (20%)	3 (25%)	<0.001
Perirenal fat invasion	28 (61%)	4 (80%)	4 (33%)	0.2
Renal vein thrombosis	18 (39%)	0 (0%)	2 (17%)	0.12
Lymphadenopathy	16 (35%)	4 (80%)	8 (67%)	0.038
Metastatic disease	19 (41%)	2 (40%)	4 (33%)	>0.9

## Data Availability

The data presented in this study are not publicly available due to privacy and ethical restrictions. Access to anonymized datasets may be provided by the corresponding author upon reasonable request and pending institutional approval.

## Abbreviations

The following abbreviations are used in this manuscript:

RCC	Renal cell carcinoma
UC	Urothelial carcinoma
CT	Computed tomography
MRI	Magnetic resonance imaging
IQR	Interquartile range
WHO	World Health Organization
